# Battery Draining Attack and Defense against Power Saving Wireless LAN Devices

**DOI:** 10.3390/s20072043

**Published:** 2020-04-05

**Authors:** Il-Gu Lee, Kyungmin Go, Jung Hoon Lee

**Affiliations:** 1 Department of Convergence Security Engineering, Sungshin University, Seoul 02844, Korea; iglee@sungshin.ac.kr; 2 Department of Computer Engineering, Pai Chai University Daejeon 35345, Korea; kyungmingo@pcu.ac.kr; 3 Department of Electronics Engineering and Applied Communications Research Center, Hankuk University of Foreign Studies, Yongin 17035, Korea

**Keywords:** Wi-Fi, wireless local area network, battery draining attack, power saving, security

## Abstract

Wi-Fi technology connects sensor-based things that operate with small batteries, and allows them to access the Internet from anywhere at any time and perform networking. It has become a critical element in many areas of daily life and industry, including smart homes, smart factories, smart grids, and smart cities. The Wi-Fi-based Internet of things is gradually expanding its range of uses from new industries to areas that are intimately connected to people’s lives, safety, and property. Wi-Fi technology has undergone a 20-year standardization process and continues to evolve to improve transmission speeds and service quality. Simultaneously, it has also been strengthening power-saving technology and security technology to improve energy efficiency and security while maintaining backward compatibility with past standards. This study analyzed the security vulnerabilities of the Wi-Fi power-saving mechanism used in smart devices and experimentally proved the feasibility of a battery draining attack (BDA) on commercial smartphones. The results of the experiment showed that when a battery draining attack was performed on power-saving Wi-Fi, 14 times the amount of energy was consumed compared with when a battery draining attack was not performed. This study analyzed the security vulnerabilities of the power-saving mechanism and discusses countermeasures.

## 1. Introduction

Recently, Internet of things (IoT) connectivity technologies have become essential in all areas of daily life, public infrastructure, industry, and business. Society has entered the IoT era of new value creation, where communication network technologies between things, and things with each other, enable them to interact [1,2]. As traditional sensor applications require tiny amounts of intermittent data transmission, they can be considered traditional sensor communication technologies [3]. However, recently emerged IoT applications have increased the requirements for high-definition video and high-quality sound, transmitting vast amounts of data, utilizing broadband service coverage, and having low power consumption and high efficiency [4,5,6]. For the existing communication methods applied to home routers and smart home appliances, additional costs are required to build smart home networks due to coverage limitations [7,8]. Furthermore, for the existing communication methods applied to industrial IoT, the data transmission rate and distance limitations cause low communication quality and energy efficiency problems [8,9]. In addition, when building an advanced metering infrastructure (AMI) and data collector unit (DCU), backhaul networks for long-distance distribution facility monitoring, factory facility detection, and diagnostic systems, due to their large number of nodes, should transmit and receive real-time data in broadband; the data rate and service coverage of existing sensor network technologies are insufficient [8,10]. Furthermore, recently emergent security cameras and Artificial Intelligence (AI)-based applications require high resolution and high computing power, and data requirements are increasing due to IoT convergence that supports various functions [11,12]. In medical healthcare smart devices, intelligence and security embedded solutions are needed for ambient assisted living [13]. Therefore, Wi-Fi technologies have attracted attention as a powerful IoT connectivity solution [14,15]. IEEE 802.11 standardization began in the 1990s, with the goal of supporting 1 Mbps transfer rates in the 2.4 GHz band for unlicensed band personal wireless applications [16]. Since then, under the name Wireless Fidelity (Wi-Fi), IEEE 802.11 has become the representative wireless communication technology protocol for accessing wireless Internet in offices, homes, airports, hotels, restaurants, trains, planes, etc., and as smart devices become ubiquitous, they have become essential in daily life [16]. The standards are currently divided into the 802.11a/b/g/n/ac/ax series that aim for ultra-fast transmissions in dense networks utilizing 2.4/5 GHz Industrial Scientific and Medical (ISM) band frequencies, and the 802.11af/ah series for IoT wide area network services in sub 1 GHz bands [14,17,18].

This study undertook a battery draining attack (BDA) on a commercial smartphone adopting IEEE 802.11a/b/g/n/ac to prove the existence of security vulnerabilities experimentally in the Wi-Fi power-saving mechanism. Similar to most technologies, when Wi-Fi’s power-saving mechanisms were introduced, they were designed and standardized with a focus on performance and QoS rather than security, and it was difficult to consider the security threats that would arise in the future when designing the technology. Wi-Fi has lasted for two decades, and its areas of use have expanded dramatically compared with those in the past, which has added to the severity of the problem. This is because Wi-Fi provides backward compatibility, indicating that the security vulnerabilities of the power-saving mechanism of the early period are still present in the later Wi-Fi standards.

Based on the technological background, this study analyzed the security vulnerabilities of the power-saving mechanism and experimentally proved that malicious attackers can exploit these vulnerabilities by continuously transmitting wake-up trigger frames during a doze mode to cause power consumption, and they can increase the efficiency of the battery draining attack by training the trigger frames.

This study makes the following three major contributions:

First, it shows the security vulnerabilities in the power-saving mechanism of the Wi-Fi standard and the validity of the power-saving security vulnerabilities by performing experiments on commercial smartphones.

Second, it analyzes the security vulnerabilities of the Wi-Fi power-saving mechanism based on experimental results in the real world and proposes countermeasures.

Third, it discusses the security remedies and presents a feasible defense technique against the battery draining attack.

The rest of this paper is organized as follows. Section 2 describes the background and discusses related works. Section 3 describes the security vulnerabilities of Wi-Fi and potential battery draining attack. Section 4 discusses the experimental setup and results. Section 5 describes and analyzes the necessary security measures. Finally, Section 6 presents the conclusions.

## 2. Background and Related Works

### 2.1. Background

Wi-Fi technology has continually evolved to improve data transmission speeds and energy efficiency [14,16]. In the case of the IEEE 802.11a/b/g/n/ac wireless local area network (WLAN) in the 2.4/5 GHz frequency band, channel bandwidth has been increased and transmission speeds have been improved by the introduction of advanced transmission technologies such as high-order modulation transmission methods, low-density parity check, and beamforming [19]. IEEE 802.11ah/af WLAN in the sub-1-GHz frequency band has greatly increased service coverage in IoT environments while simultaneously providing transmission speeds higher than those of existing wireless sensor networks [20].

To ensure high reliability and process high-speed data from long distances, systems have become more complex owing to large circuit sizes and complex signal processing procedures [21]. In addition, it has been necessary to increase the operating frequencies of digital-to-analog converters, analog-to-digital converters, and digital processors. Consequently, Wi-Fi technology has evolved, and transmission speeds and service quality have improved as the size and complexity of circuits have increased, resulting in large increases in power consumption [22,23]. However, advances in battery technology are progressing very slowly in comparison with the increases in power consumption [24].

The power consumption of digital circuits is proportional to the size of the operating circuit, the operating clock speed, and the square of the voltage [25]. Therefore, the power consumption of wireless devices can be linearly reduced by reducing the clock speed or the operating circuits. The power consumption of communication devices made with complementary metal-oxide-semiconductor (CMOS) circuits is defined as the sum of dynamic power consumption and static power consumption. Dynamic power consumption is proportional to the overall gate size and operating frequency, and it is proportional to the square of the operating voltage [26]. Static power consumption is determined by the current generated by the ground or supply current’s residual current, thermal noise, and process [27]. Here, the static power consumption is determined by the semiconductor manufacturing process and basic industrial technology, but the dynamic power consumption is determined by the system design and power-saving protocol. Therefore, the objective of a wireless communication system design is to reduce dynamic power consumption to improve the energy efficiency of the overall system.

In a station (STA) that follows the IEEE 802.11 standard, channel resources are shared by a method in which the medium access control, which is based on carrier sensing multiple access/collision avoidance (CSMA/CA), accesses the medium in a distributed contention mechanism [28]. This distributed contention-based medium access method is always monitoring the carrier signal in the medium. It receives packets, decodes the addresses, and then discards them. As such, it unavoidably consumes more power than other wireless technologies do.

Each time a new Wi-Fi standard is released, the circuit size becomes larger and the operating frequency becomes higher to improve the transmission speeds, service coverage, and quality of service (QoS) further [16]. As such, it has developed a structure in which power consumption has unavoidably increased. Wi-Fi has introduced various power-saving protocols and mechanisms to reduce dynamic power consumption and increase energy efficiency [16,28]. Consequently, it is effective in terms of performance and can be used without problems when scenarios such as battery draining attacks are not considered.

As such, great importance is placed upon the security and efficiency of the power-saving mechanisms of Wi-Fi-based IoT in environments used in fields related to human life, safety, and property. Currently, the range of uses of Wi-Fi is quickly expanding into areas intimately connected to people’s lives, safety, and property, such as smart homes, smart factories, smart grids, smart cities, vehicular networks, and medical implantable devices [29,30,31,32,33]. In the past, attacks that shut down Wi-Fi only caused harm to data availability. However, owing to the spread of IoT and the development of information communication network technology, the cyber world and the physical world are always connected, and the influence of sensors and communication devices is gradually increasing [34]. In an increasing number of cases, attacks are threatening human lives and safety or damaging property. To extend the battery lifetimes of Wi-Fi-based IoT and ensure security in terms of the availability of services related to human life, safety, and property, there is an urgent need for countermeasures based on analyses of security vulnerabilities to battery draining attacks.

### 2.2. Related Works

**Figure 1:**
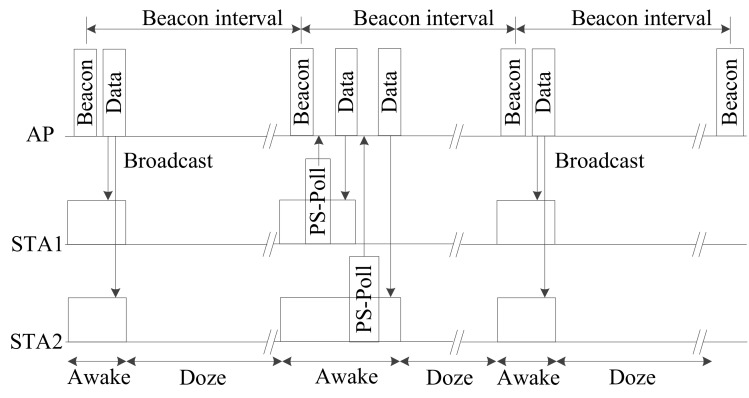
802.11 legacy power-saving mechanism.

As the Wi-Fi standard has evolved over the past 20 years, power-saving technology has been continually improved. Figure 1 shows the operating mechanism of the legacy power-saving mode (PSM) [35]. Usually, a WLAN consists of one or more access points (APs), which connect to the wired LAN, and remote client devices that connect to the AP by a wireless link. The remote devices are usually portable devices or sensors that use battery power. When the legacy PSM is used, the power-saving station wakes up at the beacon transmission period to send a periodic beacon frame during the doze mode. The traffic indication map (TIM) shown as the station’s association ID (AID) in the received beacon frame is examined to check if the traffic specified by the address is waiting to be sent in the AP buffer. After checking whether it has data to send, the station enters wake mode or doze mode. If there is no data to send in doze mode, dynamic power consumption can be reduced by gating the power supply device or system clock.

In the wake mode, wireless communication devices consume far more power than in the doze mode. Therefore, power consumption efficiency is improved by having the station transmit a power-saving poll (PS-Poll) frame to the AP, which responds with a PS-Poll response frame that includes buffered data frame information. The station uses the buffered transmission standby status frame information that was buffered in the AP to control the power-saving mode. Wi-Fi supports unscheduled automatic power save delivery (APSD) to increase energy efficiency. This power-saving feature is called Wi-Fi multi-media (WMM) [28,35]. A station that uses legacy PSM must awaken according to the beacon frame period and send/receive data, while a WMM-PS station can send/receive data at any time.

In the power save multi-poll method, stations can operate in group units. In spatial multiplexing power save, Request to Send (RTS) and Clear to Send (CTS) frames are used to activate multiple antennas. During carrier sensing in wake mode, a receiving path with only one antenna is activated, and after carrier sensing, receiving paths with all antennas are activated to improve the power consumption efficiency [36]. 

Various advanced power-saving mechanisms such as these are being introduced and used in the latest Wi-Fi standards. However, Wi-Fi ensures backward compatibility; therefore, the latest Wi-Fi device is required to support the legacy PSM from the early period of Wi-Fi. This study experimentally proves that even a wireless device that uses the latest Wi-Fi standard is vulnerable to battery draining attacks owing to this reason.

The sensors used in sensor networks have been standardized so that they can be used in low-cost, low-performance, and low-power applications. To provide these characteristics and implement such features, it is important to create lightweight designs so that the sensors are as simple as possible. Therefore, sensors were developed with structures that allow for efficient cooperation, and their power-saving mechanisms were simplified. However, low-cost, low-performance, low-power, and lightweight systems often neglect security.

Sensor networks provide simplified power-saving protocols for sensor applications using a method called S-Medium Access Control (MAC), which is different from the IEEE 802.11 MAC [37,38]. S-MAC reduces power consumption by reducing duty cycles and latency in multi-hop networks. Sensor network nodes form groups that can share sleep schedules to reduce control overhead and efficiently manage wake-up intervals according to traffic levels. However, wireless sensor nodes in a sensor network remain inactive for a long time and then are activated when a carrier is detected. During this sequential process, they cannot check other information, and they are unavoidably vulnerable to battery draining attacks because they wake up according to whether a carrier exists. It has been reported that sensor networks are vulnerable to sleep deprivation attacks (SDA) owing to their simplified power-saving methods and their traffic-dependent wake up mechanism [39,40,41].

However, IEEE 802.11 MAC defines a protocol and conditions that search the data frame buffered in the AP and wake the station that performs the power saving. Therefore, the SDAs that are effective for sensor networks are not effective for power-saving Wi-Fi. If the power management bit is set to 0 in the data frames or null frames sent to the AP by the station, the data buffered in the AP are sent, and the station receives them in wake mode. Subsequently, the station uses a different frame with a power management bit set to 1 to return to power-saving mode.

In power-saving stations that use contention-based channel access methods, the AP sends a buffered frame to the station when the station sends a PS-Poll frame. If the “More bit” field in the sent frame is set to 1, it indicates that the AP buffer has more data to send. As the most basic Wi-Fi power-saving method, legacy PSM can use TIM to determine if there are buffered data in the AP. TIM is a bitmap that shows the state of buffered data for each station associated with the AP. It is sent to the stations via the beacon transmitted as a periodic broadcast frame by the AP. The station, which is synchronized with the AP, is in a doze state and then wakes up when the beacon is sent. It receives the beacon and interprets the TIM data. The buffered data are included in the TIM data. If the “More bit” in the control field is set to 1, the station stays awake and waits to receive data [16,28].

The frame format of wireless communication devices consists of a header and a data body. The header, which includes the address information and the information for receiving data, is not encoded. This aids in efficient reception by minimizing the latency of the receiving device and makes it easy to transmit to a different node. Therefore, anybody can easily acquire the frame header information of all wireless communication devices. In the past, there was only basic information in the frame header, but recent wireless communication includes advanced information, including ID information, to support power-saving and advanced transmission features. Malicious attackers can track specific devices by extracting device fingerprint information or unprotected frame header information. It has been proven that these security vulnerabilities can be used to perform continuous jamming attacks even when the Wi-Fi channel is adjusted in dense networks [42].

## 3. Battery Draining Attack against Power-Saving WLAN Devices

The legacy power-saving mechanism of Wi-Fi technologies has two security vulnerabilities. 

First, the wireless stations switch to wake mode depending solely on the TIM bit of the beacon frame, which is unverifiable. Therefore, an attacker can keep a station awake by sniffing the beacon frames when there are buffered packets at the AP and sending a fake beacon frame. After waking the target station, the attacker can cause it to consume energy by processing fake frames. 

Second, the Wi-Fi standard defines the power-saving mechanism as being operated by the beacon frame, but actual implemented systems maintain and improve processing speed and QoS even if power saving is active by extending the wake mode so that a new frame is received at a time when a power-saving interval must be entered. Therefore, when an attacker continuously sends fake frames to an attack target, the attack target continuously extends the wake mode. Consequently, the power-saving mode cannot be activated, and severe power consumption occurs. 

**Figure 2:**
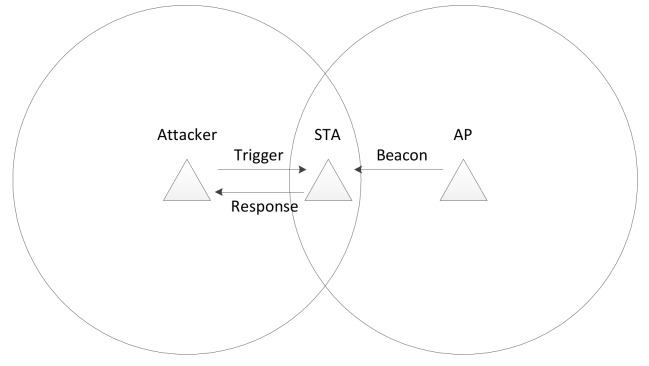
Network configuration for the battery draining attack.

Figure 2 shows the network configuration for the battery draining attack. The AP and station are associated and connected to each other. The station is in power-saving mode. It periodically receives the beacon signal from the AP and performs power saving. At this point, the attacker sends a trigger signal to the station and causes the transmission of a response frame. The station becomes the victim of the attacker as it is unable to enter the doze mode and continuously transmits response frames and consumes power.

**Figure 3:**
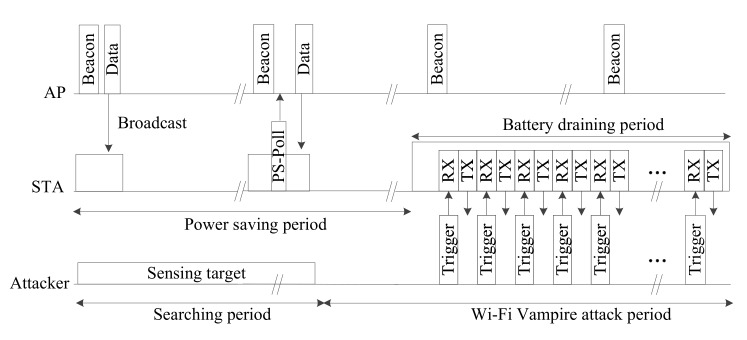
Timing diagram for the battery draining attack.

Figure 3 shows a timing diagram of the battery draining attack. As the target station performs power saving, it is periodically woken to receive the beacon frames sent by the AP, and the power-saving mechanism operates. At this point, the attacker finds the attack target station and then sends a trigger frame. When the attacker periodically sends a trigger frame, the target station is triggered and sends a response frame. The wake mode of the target is extended by this periodic operation process. If another trigger frame is sent before the extended wake mode interval is finished, the wake mode is extended. If trigger frames are sent at fixed intervals in this way, the attack target cannot enter the power-saving mode. Consequently, the target station consumes power to receive the triggering frames and send the response frames, and it cannot perform power saving for the length of the extended wake mode time. As it sends response frames from a waking state, its power consumption is increased.

## 4. Experimental Setup and Evaluation Results

**Figure 4:**
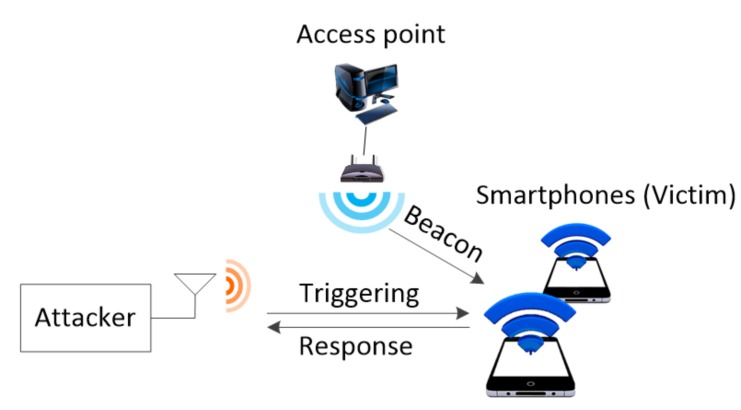
Experimental setup and network configuration.

Figure 4 shows the experimental setup and the network configuration. The experimental setup of the attacker consisted of a Wi-Fi packet generating device, commercial AP, and commercial smartphone. A field-programmable gate array was used to implement the Wi-Fi packet generating device so that it could generate a standard IEEE 802.11a/b/g/n/ac Wi-Fi signal in the 2.4/5 GHz frequency band. The commercial AP was a 2.4/5 GHz dual band wireless AP. For the commercial smartphone, a comparative analysis was performed on products from two different manufacturers, which were tested together. The Wi-Fi packet generating device performed the role of the attacker, and a software controller was used to perform a battery draining attack. The network consisted of the AP, stations, and the attacker. The stations were the commercial cellphones that were the targets of the attack. To verify the battery draining effect of the attack, the phones were set to airplane mode and then the WLAN wireless interface was turned on. The cellular radio, Bluetooth, and NFC wireless interfaces and applications were all turned off. In this study, the remaining amount of battery shown by the smartphone was used to measure the relative power consumption caused by the battery draining attack [43,44]. The test was performed when the remaining battery of the smartphone was 20%. The frame format was the IEEE 802.11a/n/ac standard format. The frame lengths were 40 bytes and 800 bytes.

**Figure 5:**
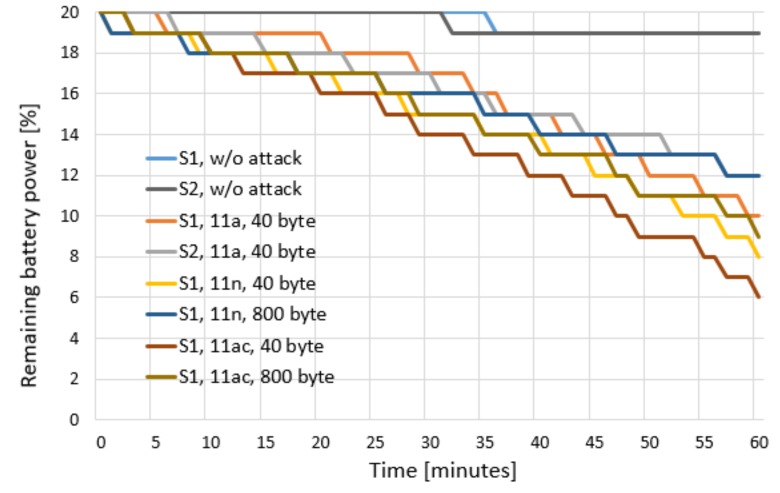
Evaluation results of the battery draining attack.

Figure 5 shows the results of the battery draining attack experiment. The experiment results show the amount of remaining battery in the smartphones made by Company S1 and Company S2 according to whether an attack occurred and the attack type. When there was no attack (w/o attack), the normal power consumption of a power-saving Wi-Fi device occurred. However, during the battery draining attack, triggering frames were used to prevent power saving from occurring. To determine the difference between manufacturers, the manufacturers S1 and S2 were compared in the w/o attack case and the 802.11a standard frame case. To determine the effect of frame length in the 802.11n and 802.11ac formats on the S1 smartphone, tests were performed with 40-byte and 800-byte frame lengths. When the attacker did not perform the attack, the station operated in the normal power-saving mode, and the power consumption efficiency was good. When the attacker began the attack, the power consumption increased significantly.

The experiments show that when the attacker periodically sent frames, the target station was triggered to send response frames even though it was using a power-saving mode. Furthermore, when the triggering frame length was shorter, the target was awakened more often, and the power consumption became more severe. When the frame size was the same, the transmission speed of 802.11ac was faster than that of 802.11n, and the number of times that the target was triggered to send response frames was greater per unit time, making the power consumption more severe. According to the experiment results, the power consumption per unit time increased by a factor of 14 when the battery draining attack was performed in this study compared with when normal power-saving occurred without an attack.

**Figure 6:**
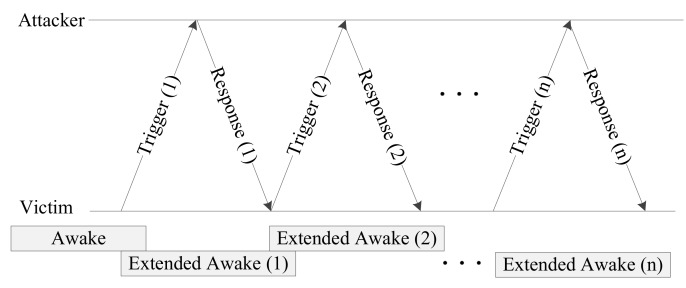
Awaken time extension analysis.

Figure 6 shows an analysis of the experiment results. Although a verification mechanism and protocol are used for the state transitions of the power-saving mechanisms, such as TIM, PS-Poll, and the “More bit,” existing commercial Wi-Fi devices are vulnerable to this battery draining attack, and the wake status can be continually extended before entering the power-saving mode. In short, the Wi-Fi power-saving security vulnerability exists so that priority can be given to processing and responding to received frames to improve performance and QoS. 

**Figure 7:**
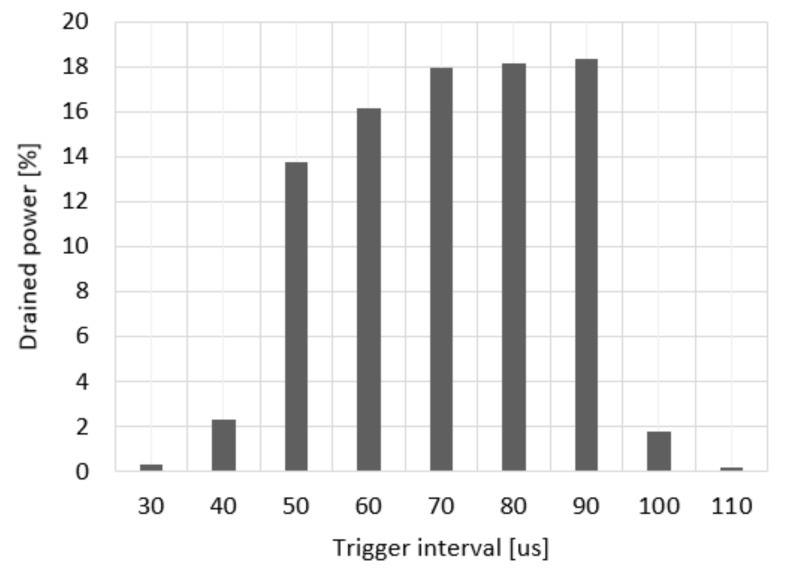
Triggering interval vs. battery drained power.

Figure 7 shows the results of the experiments on the power consumption of the attack target according to the triggering interval. The effect of the battery draining attack tested in this study can be optimized by finding a suitable triggering interval, transmission speed, and packet size, and then by adjusting the parameters and observing the response of the target. Consequently, the attacker can cause the attack target to shut down in a short amount of time. According to the experiment results, more than 14% of power was consumed when the triggering interval was set to 50–90 µs. When the triggering interval was less than 40 µs, the transmission time of the response frame overlapped with that of the next triggering frame, and therefore, the attack was not effective. When the triggering interval was greater than 100 µs, the time until the next triggering frame exceeded the time limit in which the awake time of the target can be extended, and the attack was not effective. As shown in the experiments, the attacker can observe the response frame and train the triggering frame interval to determine the suitable frame interval.

## 5. Discussions and Analysis on Security Remedies

To improve performance and QoS, Wi-Fi implements a method that extends the wake mode until the received frames are processed before entering the power-saving mode. However, this action is not always valid, and it is vulnerable to the battery draining attack, as shown in this study. Therefore, this paper proposes the incorporation of two security measures to prevent the battery draining attack on power-saving Wi-Fi.

First, power-saving devices are implemented so that the wake state can be extended by RTS or a data frame, and they periodically switch between wake mode and doze mode to perform power saving. These devices can be implemented so that the wake mode is maintained only by beacon frames. If they are implemented thus, direct battery draining attacks will become difficult. Attacks will be difficult to perform because they must be performed through the AP, or rogue AP attacks must be performed.

Second, the station should extend the wake mode only when a shared secret key in the received frame is checked and observed to match, and not simply when a frame arrives or a certain field value has been set. If the shared secret key does not match, the station changes from power-saving mode to sleep mode. If attacks are frequent, it enters a deep sleep and does not respond to triggering signals for a long time.

**Figure 8:**
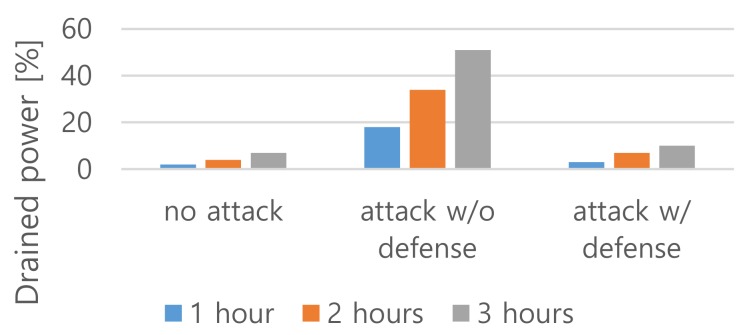
Evaluation results on the Wi-Fi power switching method.

However, the above two methods have the problem of either revising the existing standards or modifying the implemented chipsets. To improve the security of the existing Wi-Fi chipsets that contain the vulnerabilities to the battery draining attacks, the Wi-Fi power switching method was tested in this study. The Wi-Fi power switching method can be applied with either a firmware or software level patch even if a traditional chipset is used. This is because if a repeat triggering frame is detected, the Wi-Fi function can be switched off and the attack can be monitored again after the power-off duration. The power-off duration can be increased several times or exponentially depending on the number of repeated detection of attacks. If a battery draining attack is detected by monitoring the repeated triggering frames, it is designed to prevent the worst-case scenario when the main system is shut down by powering off. The Wi-Fi power switching method powers off only the Wi-Fi functions and maintains other activities in the system. In the Wi-Fi power-off state, the main system stores the data in memory. When it needs to transfer the stored data to the AP, it powers on and transfers the stored data. Figure 8 shows the defense efficiency against a battery draining attack when the Wi-Fi power switching method is applied. In this experiment, 11ac mode 40-byte length frames with a trigger interval 80 µs were applied for attack and measured the drained power at intervals of one to three hours with a one-hour step. In this test, the power-off state duration was increased by 2^*n* minutes, where *n* is the cumulative number of attack detections.

**Figure 9:**
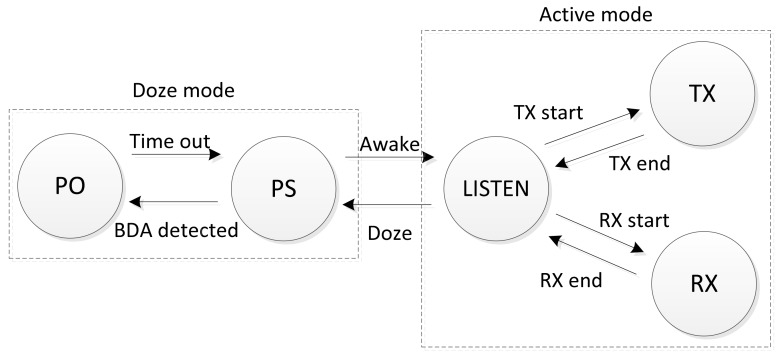
Wi-Fi power state diagram.

Figure 9 shows the power state diagram for the proposed Wi-Fi power switching method. There are five states of Wi-Fi devices. The TX, RX, and LISTEN states are parts of the active mode, and the power-save (PS) and power-off (PO) states are parts of the Wi-Fi doze mode. In the active mode, the wireless device is in an awake state and actively transmits/receives data with the access point. If the wireless device determines it is not being paged, it enters the power-save state. Otherwise, it enters the awake state for a certain period of time to receive the data frames. The Wi-Fi power switching method turns the Wi-Fi radio off when a battery draining attack (BDA) is detected.

The conventional Wi-Fi power saving method changes the sleep mode and awake mode periodically to receive the beacon signal sent by the AP for determining whether it will remain awake or not. On the other hand, for defense against the battery draining attack, the Wi-Fi power switching method powers on only when the uplink transmission is required, and downlink transmission is not supported. Assuming a single channel condition, there is a trade-off relationship between security against battery draining attacks and downlink performance for link throughput. However, if the Wi-Fi power switching method is used in an environment where multiple channels are available, the downlink performance loss can also be prevented by channel switching if the draining attack persists during power-on. In some cases, as in the [42] paper, there may be persistent attacks by device tracking, which requires further study of these advanced attacks.

## 6. Conclusions

In the IoT era, Wi-Fi has a wide range of uses intimately connected to people’s lives, safety, and property, such as smart homes, smart grids, smart cities, connected cars, traffic infrastructure, and medical implantable devices. Wi-Fi was been developed with backward compatibility, and the impact and power of its vulnerabilities have become stronger during its evolution. As such, security for battery life and the power-saving mechanisms of Wi-Fi applications has become a factor of primary importance. This study performed a battery draining attack on the power-saving Wi-Fi of existing commercial smartphones and analyzed the limitations of existing Wi-Fi power-saving technology and implementations. 

The security vulnerabilities demonstrated in the experiments in this study occurred because the power-saving protocol was designed and implemented with a focus on performance and QoS. To resolve the vulnerabilities, it is possible to perform safe power-saving by limiting the types of frames that can extend the wake mode and verifying a shared secret key to determine whether the wake mode will be extended if the standards and implemented design are modified. Otherwise, it is possible to adopt the fetched version including defense mechanisms such as a Wi-Fi power switching scheme.

Future studies will use mathematical and theoretical modeling to simulate and analyze battery draining attacks and defenses, and they will use various battery draining attack scenarios to verify the validity of the defense method proposed in this paper.

## Abbreviations

The following abbreviations are used in this manuscript:

IoT	Internet of things
AMI	Advanced Metering Infrastructure
DCU	Data Collector Unit
AI	Artificial Intelligence
Wi-Fi	Wireless Fidelity
ISM	Industrial Scientific and Medical
CMOS	Complementary Metal-Oxide-Semiconductor
WLAN	Wireless Local Area Network
STA	Station
CSMA/CA	Carrier Sensing Multiple Access/Collision Avoidance
QoS	Quality of Service
PSM	Power Saving Mode
AP	Access Point
TIM	Traffic Indication Map
AID	Association ID
PS-Poll	PS-Poll
ASPD	Automatic Power Save Delivery
WMM	Wi-Fi Multi-Media
RTS	Request To Send
CTS	Clear To Send
MAC	Medium Access Control
